# Factors contributing to measles transmission during an outbreak in Kamwenge District, Western Uganda, April to August 2015

**DOI:** 10.1186/s12879-017-2941-4

**Published:** 2018-01-08

**Authors:** Fred Nsubuga, Lilian Bulage, Immaculate Ampeire, Joseph K. B. Matovu, Simon Kasasa, Patricia Tanifum, Alex Ario Riolexus, Bao-Ping Zhu

**Affiliations:** 1 Uganda Public Health Fellowship Program – Field Epidemiology Track, Makerere University School of Public Health, P.O. Box 7072, Kampala, Uganda; 2 Uganda National Expanded Program on Immunization, Ministry of Health, Kampala, Uganda; 3 Makerere University School of Public Health, Kampala, Uganda; 4 Division of Public Health Protection, Center for Global Health, US Centers for Disease Control and Prevention, Kampala, Uganda

**Keywords:** Measles, Vaccine effectiveness, Vaccine failure, Case-control study, Global health security

## Abstract

**Background:**

In April 2015, Kamwenge District, western Uganda reported a measles outbreak. We investigated the outbreak to identify potential exposures that facilitated measles transmission, assess vaccine effectiveness (VE) and vaccination coverage (VC), and recommend prevention and control measures.

**Methods:**

For this investigation, a probable case was defined as onset of fever and generalized maculopapular rash, plus ≥1 of the following symptoms: Coryza, conjunctivitis, or cough. A confirmed case was defined as a probable case plus identification of measles-specific IgM in serum. For case-finding, we reviewed patients’ medical records and conducted in-home patient examination. In a case-control study, we compared exposures of case-patients and controls matched by age and village of residence. For children aged 9 m-5y, we estimated VC using the percent of children among the controls who had been vaccinated against measles, and calculated VE using the formula, VE = 1 - OR_M-H_, where OR_M-H_ was the Mantel-Haenszel odds ratio associated with having a measles vaccination history.

**Results:**

We identified 213 probable cases with onset between April and August, 2015. Of 23 blood specimens collected, 78% were positive for measles-specific IgM. Measles attack rate was highest in the youngest age-group, 0-5y (13/10,000), and decreased as age increased. The epidemic curve indicated sustained propagation in the community. Of the 50 case-patients and 200 controls, 42% of case-patients and 12% of controls visited health centers during their likely exposure period (OR_M-H_ = 6.1; 95% CI = 2.7–14). Among children aged 9 m-5y, VE was estimated at 70% (95% CI: 24–88%), and VC at 75% (95% CI: 67–83%). Excessive crowding was observed at all health centers; no patient triage-system existed.

**Conclusions:**

The spread of measles during this outbreak was facilitated by patient mixing at crowded health centers, suboptimal VE and inadequate VC. We recommended emergency immunization campaign targeting children <5y in the affected sub-counties, as well as triaging and isolation of febrile or rash patients visiting health centers.

## Background

Measles is one of the most infectious human diseases and frequently results in widespread outbreaks. It can lead to lifelong complications and death [1, 2]. The World Health Organization (WHO) estimated that approximately 535,000 children died of measles in 2000 globally, the majority from developing countries, which accounted for 5% of all under-five mortality [3]. In 2009, the Regional Committee for Africa adopted a regional measles elimination goal for 2020 at its 59th session. It urged member states to invest in strengthening immunization and health systems, because routine immunization plays a central role in the elimination efforts [4]. In 2010, the World Health Assembly set a Year 2015 Target to reduce measles deaths by 95% of the 2000 levels. By 2010, global measles mortality decreased by an estimated 74%, from 535,300 deaths in 2000 to 139,300 in 2010 [5].

Accelerated measles control activities started in 2001 in countries in the WHO African Region [6]. By 2008, reported measles cases decreased by 93% and estimated measles mortality decreased by 92% in the African Region compared with the figures for 2000 [6]. The WHO African Region set targets as part of the regional measles elimination goal, which include the following: Reducing annual regional measles incidence to fewer than five cases per million; achieving measles vaccination coverage (VC) of 90% nationally and exceeding 80% VC at every districts; and achieving at least 95% coverage with measles vaccines during Supplementary Immunization Activities nationally and in at least 80% of districts.

Despite the effort and progress made, measles incidence appears to have rebounded in recent years in Uganda. Kamwenge District in western Uganda reported an increasing number of measles cases since April 2015. In June 2015 the district requested assistance to control the outbreak. Measles VC (with 1 dose measles-containing vaccine given at age 9 m) in Kamwenge District is estimated at 80% based on the administrative data, which does not provide adequate population protection.

Kamwenge District (0.2258° N, 30.4818° E) has an estimated total population of 421,470. It is bordered by Kyenjojo District to the north, Kyegegwa and Kiruhura Districts to the northeast, Ibanda District to the east and southeast, Rubirizi District to the southeast, Kasese District to the west, and Kabarole District to the northwest. The district also houses a refugee settlement, with an estimated refugee population in excess of 50,000, a vulnerable population often with low VC, inadequate access to care, and compromised health status.

We conducted an investigation in Kamwenge District to identify potential exposures for measles transmission, estimate vaccine effectiveness (VE), estimate VC, and provide evidence-based recommendations for measles control in Uganda.

## Methods

### Case definitions

We defined a probable case as onset of fever and generalized maculopapular rash in a resident of Kamwenge from 1 March to 31 August 2015 with at least one of the following symptoms: Coryza, conjunctivitis, or cough. A confirmed case was a probable case with serum positivity of measles-specific IgM antibody. We developed the case definition after reviewing the clinical presentations of some of the measles patients and discussing with clinicians.

### Case finding

We conducted systematic case finding by visiting health centers that served the most affected sub-counties. We reviewed patient records from 1 March 2015 to 31 August 2015 to identify probable and confirmed cases based on the case definition. The surveillance officers, village health team (VHT) members and village administrators visited the case-patients’ homes to verify the cases. The records in the Health Management Information System had basic information for each patient, including name, age, sex, residence, admission date, and symptoms. We trained other health workers and VHT members on case finding using the case definition. Those meeting the definition of a probable case were referred to health centers for further management. The VHT members played a key role during this process because they reside in the villages and know practically every village resident, and they are trained to conduct surveillance of notifiable diseases and to refer patients for health services.

### Descriptive epidemiologic analysis

We conducted a descriptive epidemiologic study examining the distribution of the cases. We described the clinical symptoms of the case-patients. We computed the attack rates by age, sex, and sub-county of residence, and nationality. We constructed an epidemic curve to examine the development of the epidemic over time.

### Hypothesis generation

During hypothesis generation we used a standardized case investigation form to interview 24 probable case-patients who were conveniently found at the health facilities and surrounding communities. The assumption was that those probable case-patients represented all case-patients during this outbreak. The sample size was not statistically estimated formally but it was based on experiences during past outbreak investigations. These interviews explored potential exposures at health facilities, schools, churches, and during any mass vaccination campaigns within the last 21 days before onset of rash.

### Case-control study

We conducted a matched case-control study to evaluate the potential exposures that emerged during hypothesis generation. Cases for the case-control study were selected among the probable cases identified through case finding. If a household had more than one case, only the case-patient with the earliest onset was enrolled.

For each case, we selected four controls, individually matched with the case by age group (0–5, 6–12, 13–18 and 19–30) and village of residence. We matched by age because age is a major confounder during investigations of virtually all communicable diseases. We matched by village of residence to ensure cases and controls had comparable probability of exposure. We used a case-to-control ratio of 1:4 because little additional statistical power is gained beyond four controls per case [7]. A control-person must have had no fever nor generalized maculopapular rash since March 2015 to qualify.

We administered a structured questionnaire to cases and controls in-person, to collect information on demographic characteristics (age, sex, and education level), potential exposures, and vaccination status. In interviewing the cases and controls about their potential exposures, we defined the effective exposure period to be the time window during 7–21 days prior to the case-patients’ onset of rash (i.e., between the minimum and maximum incubation period for measles). These calendar days were then applied in interviewing both the case-patient and his/her matched control-persons regarding their exposures. Cases that had rash onset between 30 April and 13 July 2015 were recruited for the case-control study.

Vaccination history was assessed by a vaccination card whenever available; for 24% (12/50) of the case-patients and 13% (25/200) of the control-persons whose vaccination cards were unavailable, we relied on the recall of the case-patients (for adult cases) or their parents (for child cases).

### Data management and analysis

We managed the data using Microsoft Excel, and conducted data analysis using Epi Info 7.1.5. We used the 2014 census data to calculate attack rates. We used the Mantel-Haenszel method to analyze the data from the case-control study to account for the matched study design.

We estimated the VE for measles vaccine using the following formula [8]:

$$ \mathrm{VE}=\left(1-{\mathrm{OR}}_{\mathrm{M}-\mathrm{H}}\right) $$

where OR_M-H_ is the protective Mantel-Haenszel odds ratio associated with vaccination estimated from the case-control study. We estimated the VE for all persons aged ≥9 m and by age group (9 m-5y, 6-12y, and 13-52y). We excluded children <9 m of age from the calculation of the VE because Uganda’s routine one-dose measles vaccination is administered at age 9 m.

To obtain a quick estimate of VC in the outbreak area for the purpose of outbreak control, we used the percentage of controls who had a history of measles vaccination to estimate measles VC for all persons aged ≥9 m and by age groups (9 m-5y, 6–12, and 13-52y), assuming that the controls were representative of the general population [9, 10]. As with the calculation of VE, children aged <9 m were excluded in calculating VC.

### Laboratory and environmental investigations

We collected blood samples from 23 probable case-patients who sought care at health centers and sent the samples to the Uganda Virus Research Institute for serological testing. Measles IgM antibody levels were measured using enzyme linked immunosorbent assay (ELISA) kits.

During the case-finding activities, we conducted on-site inspections of the health centers in the outbreak area to observe the crowdedness, and interviewed the clinic attendants on the reasons for the crowdedness.

### Ethical considerations

The Ministry of Health of Uganda (MoH) gave the directive and approval to investigate this outbreak. The Office of the Associate Director for Science, CDC/Uganda, determined that this activity was not human subject research, and its primary intent was public health practice or a disease control activity (specifically, epidemic or endemic disease control activity). Verbal informed consent was obtained from all patients who provided blood samples and from all participants or, caretakers (if the interviewee/patient was a minor), before the start of each interview. We sought verbal consent because this study was conducted as part of an outbreak investigation whose primary purpose was to inform disease control efforts rather than outright human subjects’ research.

## Results

**Fig. 1:**
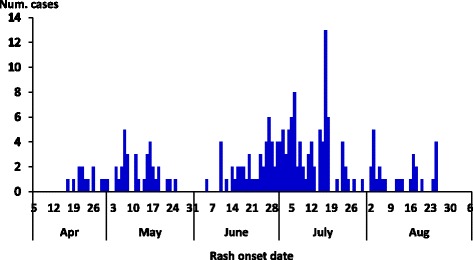
Epi curve showing the number of measles cases by rash onset date in Kamwenge District, from April to August 2015

We identified 213 probable cases with onset between 17 April and 30 August, 2015 in the three affected sub-counties, including 18 laboratory confirmed cases. The epidemic curve shows sustained community transmission from April to August with no apparent periodicity. The highest number of cases had onset in July (Fig. 1).

The median age of the case-patients was 5y (Inter-Quartile Range: 2.3–9.5y). The clinical presentations of case-patients’ were consistent with measles (Table 1). The attack rate was highest in children aged 0-5y (13 per 10,000) and declined as age increased. Of the three sub-counties, Biguli (where the outbreak started) had the highest attack rate. Males and females were relatively equally affected. The attack rate also did not differ greatly between Ugandan nationals and refugees (Table 2).

**Table 1:** Distribution of symptoms of measles case-patients during an outbreak in Kamwenge District, Uganda, April to August 2015

Clinical features	% (Total *n* = 213)
Fever	100
Maculopapular rash	100
Cough	81
Coryza	48
Conjunctivitis	45

**Table 2:** Attack rates of measles (per 10,000) during an outbreak in Kamwenge District, Uganda, April to August 2015

Characteristic	Frequency	Population	Attack rate(/10,000)
Age group			
0–5	112	83,788	13
6–12	56	77,622	7.2
13–60	39	191,879	2.0
Sub-county			
Biguli	130	34,560	38
Nkoma	64	32,811	20
Bwizi	19	30,482	6.2
Sex			
Male	111	205,802	5.3
Female	102	215,668	4.7
Nationality			
Ugandan	174	381,734	4.6
Refugee	39	57,473	6.8

The hypothesis-generating interviews identified three potential exposures that might have driven the outbreak, i.e., exposures at school, at health facilities, and at the church. The case-control investigation of those potential exposures showed that visiting a health center during the effective exposure period was associated with an increased risk of developing measles by approximately 6 fold (OR_M-H_ = 6.1, 95% CI: 2.7–14) (Table 3). The other potential exposures were not significantly associated with measles disease.

**Table 3:** Exposures for measles transmission during an outbreak in Kamwenge District, Uganda, April to August 2015

Exposure factors during case-patient’s effective exposure period^a^	Number		%		OR_M-H_ (95% CI)
	Cases (*n* = 50)	Control (*n* = 200)	Cases	Control	
Visit to health center					
Yes	21	23	42	12	6.1 (2.7–14)
No	29	177	58	88	Ref
Attending school					
Yes	16	57	32	29	1.4 (0.52–4.0)
No	34	143	68	71	Ref
Attending church					
Yes	29	127	58	64	0.75 (0.37–1.5)
No	21	73	42	36	Ref

^a^Defined as the time period between 7 and 21 days prior to the case-patient’s onset of symptoms

On-site observation of health centers’ out-patient department revealed that the patient waiting areas were grossly overcrowded. Patients saturated the waiting areas most of the time during the day. Interviews of health center administrators indicated that the overcrowding was due to delays in consultation and disposition as a result of inadequate healthcare workers or an influx of patients.

Measles vaccine administered at age 9 m by the routine vaccination schedule in Uganda was protective against measles infection for the population aged ≥9 m (OR_M-H_ = 0.36, 95% CI = 0.75–0.83), with a corresponding VE of 64% (95% CI = 17–85%). When the data were stratified by age, the vaccine was protective in children aged 9 m-5y (OR_M-H_ = 0.30, 95% CI = 0.12–0.76), yielding a VE of 70% (95% CI = 24–88%). The VE for the other age groups were not statistically significant due to small sample sizes (Table 4).

**Table 4:** Measles vaccination effectiveness by age group during an outbreak in Kamwenge District, Uganda, April to August, 2015

Age (y) and Vaccination status	Number		%		OR_M-H_ (95% CI)	Vaccine Effectiveness (95% CI)
	Cases (*n* = 50)	Controls (*n* = 200)	Cases	Control		
All ages (9 m-52y)						
Vaccinated	25	127	61	77	0.36 (0.15–0.83)	64 (17–85)
Not vaccinated	16	37	39	23		
9 m-5y						
Vaccinated	16	91	52	75	0.30 (0.12–0.76)	70 (24–88)
Not vaccinated	15	30	48	25		
6-12y						
Vaccinated	4	18	80	75	0.33 (0.02–5.3)	67 (0–98)^a^
Not vaccinated	1	6	20	25		
13-52y						
Vaccinated	5	18	100	95	-^b^	-^b^
Not vaccinated	0	1	0	5		

^a^The upper bound of the 95% confidence interval for OR was >1, therefore the lower bound of the 95% confidence interval for Vaccine Effectiveness was set to 0

^b^OR and Vaccine Effectiveness for persons aged ≥13y could not be calculated because all cases were vaccinated, resulting in a zero in the denominator

The VC, estimated by the percent of control-persons who had a history of measles vaccination, was 77% for all persons age ≥ 9 m, 75% among children aged 9 m-5y, 75% among children aged 6-12y, and 95% among persons aged ≥13y (Table 5).

**Table 5:** Measles vaccination coverage by age group, estimated by the percent of controls who were vaccinated, during an outbreak in Kamwenge District, Uganda, April to August, 2015

Age group	Total^a^	# vaccinated	Vaccination coverage (%)	95% CI
All ages (9 m-52y)	164	127	77	70–84
9 m-5y	121	91	75	67–83
6-12y	24	18	75	53–90
13-52y	19	18	95	74–100

^a^20 control-persons were not included because they had unknown vaccination status

## Discussion

Our investigation indicated that exposure at crowded healthcare facilities, vaccine failure, and failure to vaccinate all facilitated measles transmission during the measles outbreak in Kamwenge District, Western Uganda.

Measles is an extremely infectious disease [11]. When a measles case is introduced into a naïve population, 12–40 secondary cases might be produced [12]. Measles virus has the ability to remain viable for an extended period of time in small droplets expelled by infected individuals when they cough, hence the disease can be easily transmitted from a measles patient to other patients if they share the same confined space such as a waiting area in a healthcare setting, especially if the waiting area is crowded [13]. Similar observations have been made in other countries [14–16]. Therefore, reducing healthcare-associated transmission should be an integral and important part of measles control strategy [17, 18]. During measles outbreaks, healthcare centers could consider setting up a triaging system to separate patients with fever and rash from other patients at the reception area. Public health authorities could also consider setting up special measles clinics and advise anyone with fever and rash to go to these special clinics for treatment.

The effectiveness of a single-dose measles vaccine currently administered at age 9 m in the outbreak area during this investigation (64% in all persons and 70% among children aged 9 m-5y) was lower than a previous estimate in three large hospitals in Dhaka, Bangladesh (80%) [19]. A literature review found that VE varied by WHO region, with lower estimates in countries belonging to the African Region and the Southeast Asian Region [20]. The effectiveness of measles vaccination is influenced by several host and vaccine factors [21], including the number of doses given, age at which vaccine is administered [22], the quality of vaccine and the adequacy of the cold chain [23, 24]. It should also be noted that in outbreak situations, usually only a small percentage of the cases are confirmed; hence researchers have to use observational study designs to estimate VE, which tend to underestimate the true VE due to the inclusion of false positives in the cases. Case-control designs usually produce the highest and most accurate estimates because the use of odds ratio tends to over-estimate the relative risk, which counterbalances the underestimation of VE due to the inclusion of false-positives in the cases [25]. Estimating VE is an important part of an outbreak investigation involving a vaccine-preventable disease because it can provide crucial evidence to guide outbreak response and routine immunization activities [20].

Measles antibodies develop in approximately 85% of children vaccinated at age 9 m, 95% of children vaccinated at 12 m, and 98% of children vaccinated at 15 m. WHO recommends vaccination at age 9 m of age in countries at the mortality reduction stage [26]. However, to improve the effectiveness of measles vaccine and to slow the buildup of the susceptible population, one or a combination of the following strategies is generally recommended, depending on the specific situation: introducing a second dose in the vaccination schedule, increasing the age of measles vaccination to ≥12 m for the first dose, and conducting supplemental immunization activities [24]. In the Ugandan situation, it would be best to introduce a second dose of measles vaccine into routine vaccination schedule at age 15 m. Literature has shown that a second dose of measles vaccine would boost the immunity level in the vaccinated population to as high as 98% and reduce the buildup of the susceptible population [27].

The VC estimated in this investigation (75%) in children 9 m-5y was lower than that from the administrative data of Kamwenge District (79%), both of which were lower than the recommended VC of ≥90% required to achieve population immunity by the WHO African Region [6]. Even in countries where good immunization coverage has been achieved, measles outbreaks still occur because susceptible population still accumulates fairly rapidly even with high immunization coverage, as measles vaccine is not 100% effective [28]. For example, despite having an overall measles vaccination coverage of 92–94% between 2004 and 2010, WHO/Euro experienced several outbreaks; in 2010 the region reported 30,639 cases, the highest since 2006 [29]. Also, a measles outbreak occurred in San Diego, California in 2008, despite having a high community vaccination coverage at 91% [30]. The low VC found in our study therefore necessitates the Uganda MoH to regularly conduct coverage surveys to complement administrative data so as to improve program implementation.

### Strength and limitations

Our investigation used a rigorous epidemiologic approach to describe the roles of exposure at crowded healthcare facilities, vaccine failure, and failure to vaccinate during a measles outbreak. On the other hand, our study had multiple limitations. Some of the cases and controls lacked immunization cards. In Uganda, measles vaccination is the last antigen given on the upper left arm at age 9 m during routine immunization; therefore we asked the caretakers whether their children had received a measles shot at age 9 m on the upper left arm. We relied on the respondents’ memory to recall the vaccination status. Also, formal contact tracing was impossible to implement in our setting; therefore we might have had an incomplete case count, which prohibited us from examining the role of community transmission. Additionally, we used the proportion of control-persons vaccinated in the case-control study to estimate the measles VC, in order to provide data quickly to MoH for rapid outbreak control. While similar methods have been used previously in outbreak investigation settings [9, 10], this method assumed that the control-persons in the case-control study represented the general population, which might have been an invalid assumption and could have led to a biased estimate. A more appropriate method would have been a population sample survey. Lastly, use of probable cases instead of confirmed cases might have underestimated the VE in this study.

## Conclusions

We conclude from our investigation that exposure at crowded health centers, along with low VC and suboptimal VE, facilitated the spread of measles during this outbreak. We recommend emergency immunization campaign targeting children ≤5y in affected sub-counties and triaging and isolating febrile or rash patients at health centers during measles outbreaks to control the current outbreak. We also recommend that measles routine immunization schedule be changed from the current one dose at age 9 m to two doses at 9 m and 15 m each.

## Abbreviations

CDC: US Centers for Disease Control and Prevention
CI: Confidence interval
OR_adj_: Adjusted odds ratio
VE: Vaccine effectiveness
WHO: World Health Organization
